# Task-General Efficiency of Evidence Accumulation as a Computationally Defined Neurocognitive Trait: Implications for Clinical Neuroscience

**DOI:** 10.1016/j.bpsgos.2021.02.001

**Published:** 2021-03-13

**Authors:** Alexander Weigard, Chandra Sripada

**Affiliations:** Department of Psychiatry, University of Michigan, Ann Arbor, Michigan

**Keywords:** Cognitive control, Diffusion model, Executive function, Linear ballistic accumulator, Mathematical psychology, Transdiagnostic risk

## Abstract

Quantifying individual differences in higher-order cognitive functions is a foundational area of cognitive science that also has profound implications for research on psychopathology. For the past 2 decades, the dominant approach in these fields has been to attempt to fractionate higher-order functions into hypothesized components (e.g., inhibition, updating) through a combination of experimental manipulation and factor analysis. However, the putative constructs obtained through this paradigm have recently been met with substantial criticism on both theoretical and empirical grounds. Concurrently, an alternative approach has emerged focusing on parameters of formal computational models of cognition that have been developed in mathematical psychology. These models posit biologically plausible and experimentally validated explanations of the data-generating process for cognitive tasks, allowing them to be used to measure the latent mechanisms that underlie performance. One of the primary insights provided by recent applications of this approach is that individual and clinical differences in performance on a wide variety of cognitive tasks, ranging from simple choice tasks to complex executive paradigms, are largely driven by efficiency of evidence accumulation, a computational mechanism defined by sequential sampling models. This review assembles evidence for the hypothesis that efficiency of evidence accumulation is a central individual difference dimension that explains neurocognitive deficits in multiple clinical disorders and identifies ways in which this insight can advance clinical neuroscience research. We propose that recognition of efficiency of evidence accumulation as a major driver of neurocognitive differences will allow the field to make clearer inferences about cognitive abnormalities in psychopathology and their links to neurobiology.

The study of individual differences in performance on laboratory cognitive tasks and the neural basis of these differences has been a pillar of biological psychiatry research over the past several decades. This work is driven by the consistent observation that impairments in executive functions and cognitive control (hereafter referred to as higher-order cognition) are observed transdiagnostically across multiple mental disorders, including schizophrenia, externalizing disorders (attention-deficit/hyperactivity disorder [ADHD], substance use) (1, 2, 3, 4), depression, and anxiety (5,6). Links between deficits in higher-order cognition and psychopathology have prompted a swell of clinical neuroscience research aimed at better understanding their psychological and neurobiological basis (10, 11, 12, 13, 14, 15, 16, 7, 8, 9). Moreover, this work is heavily emphasized in major funding agency initiatives, such as the Research Domain Criteria project (17) and Computational Psychiatry Program (18).

Our aim in this review is to offer a critical perspective on the current state of the science; we identify a set of interrelated obstacles that have arisen for current approaches and lay out the case for an alternative framework. The Fractionation Paradigm and Recent Challenges section reviews the dominant fractionation paradigm, which aims to use factor analysis to break cognitive functions into constituent elements with selective relations to clinical disorders, and details recent findings that present serious challenges for this approach. The next three sections introduce an alternative paradigm based on computational modeling, specifically focusing on efficiency of evidence accumulation (EEA), a central individual difference dimension measured in sequential sampling models (SSMs) of cognition. We review evidence that EEA is a primary driver of individual and clinical differences in cognitive performance across a broad array of ostensibly quite distinct cognitive tasks and exhibits several advantages over metrics derived from the fractionation paradigm. Finally, we highlight key implications of this framework for clinical neuroscience.

## The Fractionation Paradigm and Recent Challenges

The dominant approach toward studying individual differences, and by extension clinical differences, in higher-order cognition involves fractionation. This framework assumes that higher-order cognition consists of multiple component functions and that each constitutes a relatively distinct individual difference dimension. This latter assumption is especially relevant to clinical neuroscience research, where it is common to postulate that disorders involve selective impairments in specific functions.

A primary tool for fractionation involves batteries of carefully constructed experimental tasks that are intended to selectively engage specific functions. For example, in the incongruent condition of the Stroop task (19), participants must respond as to the ink color of a word while ignoring the word’s semantic meaning, which indicates a discrepant color. This discrepancy is thought to engage an inhibition process that suppresses the dominant tendency to provide the (incorrect) word response. In an otherwise-similar congruent condition, in which the color of the word and its meaning are matched, it is assumed that the inhibition process is unengaged. Performance differences between the two conditions are thus assumed to precisely index individuals’ inhibition. Tasks such as the Stroop are often paired with factor analysis to study patterns of covariance across task batteries. Foundational work by Miyake *et al.* (20) yielded evidence for three core executive dimensions—response inhibition, task switching, and working memory updating—and this framework remains the most influential fractionation taxonomy [e.g., (21,22)].

A growing body of findings, however, presents serious challenges for the fractionation approach. First, in a systematic review, Karr *et al.* (23) provided evidence that many factor models in this literature were overfit to underpowered samples and that alternative models that contradict the foundational three-factor structure may be more plausible. A second challenge for this paradigm concerns fundamental psychometric properties of widely used tasks that utilize difference scores to selectively index higher-order functions (e.g., the Stroop). Such measures consistently demonstrate poor test-retest reliability (24, 25, 26, 27); that is, the rank order of subjects fails to be preserved across testing occasions, limiting the usefulness of these metrics for individual differences research (28,29). The same measures also have poor predictive validity; recent well-powered studies show that they have tenuous relationships with relevant criterion variables, such as self-regulation questionnaires (30, 31, 32, 33). A third challenge for fractionation is the failure of disorder specificity, the idea that selective executive deficits could help establish boundaries between disorders. Researchers have long sought to selectively link deficits in working memory to schizophrenia (34, 35, 36), behavioral inhibition to ADHD (37, 38, 39), and inhibition of negative thoughts to anxiety and depression (40, 41, 42). However, such selectivity has been elusive. People with psychiatric disorders, including schizophrenia, bipolar disorder, and ADHD, typically exhibit diverse cognitive impairments that cut across the higher-order domains fractionation researchers seek to distinguish (3,15,43, 44, 45, 46, 47, 48, 49).

None of these challenges to the fractionation paradigm are necessarily decisive, but they are serious enough that alternative approaches, the subject to which we now turn, deserve greater attention.

## Mathematical Psychology, Computational Psychiatry, and SSMs

Multiple recent commentaries in psychiatry, clinical psychology, and the broader behavioral sciences (50, 51, 52, 53) have highlighted a critical paradox: these fields have largely eschewed the use of formal mathematical process models, despite the substantial advancements in precision, theory development, and cumulative knowledge that such models have provided for other sciences. One notable exception is the subfield of mathematical psychology, which has a long tradition of using formalisms to specify, and stringently test, theories about the mechanisms behind cognitive processing (54, 55, 56). Beyond the general scientific advantages of mathematical modeling, including allowing greater explanatory clarity and stronger empirical tests of theoretical predictions (52,53,55), this approach has recently shown unique promise for identifying links between human cognition and neural functioning (57, 58, 59). Furthermore, mathematical psychology’s models are beginning to play a pivotal role in the emerging field of computational psychiatry, where they are used to identify candidate biobehavioral dimensions linked to psychopathology that may have clearer relationships with neurobiological mechanisms than existing cognitive constructs (50,60,61).

SSMs (62) are a prime example of computational frameworks from mathematical psychology that are now seeing wide application in the neurosciences and psychiatry. Although models in this class were originally developed to explain recognition memory and simple perceptual decisions (62, 63, 64), they have been successfully applied to a variety of complex behavioral domains (65, 66, 67), including executive tasks (68, 69, 70, 71, 72). For tasks in which individuals must choose between response options, SSMs assume that they gradually accumulate noisy evidence for each option from the environment over time until evidence for one option reaches a critical threshold, which initiates the corresponding response.

**Figure 1:**
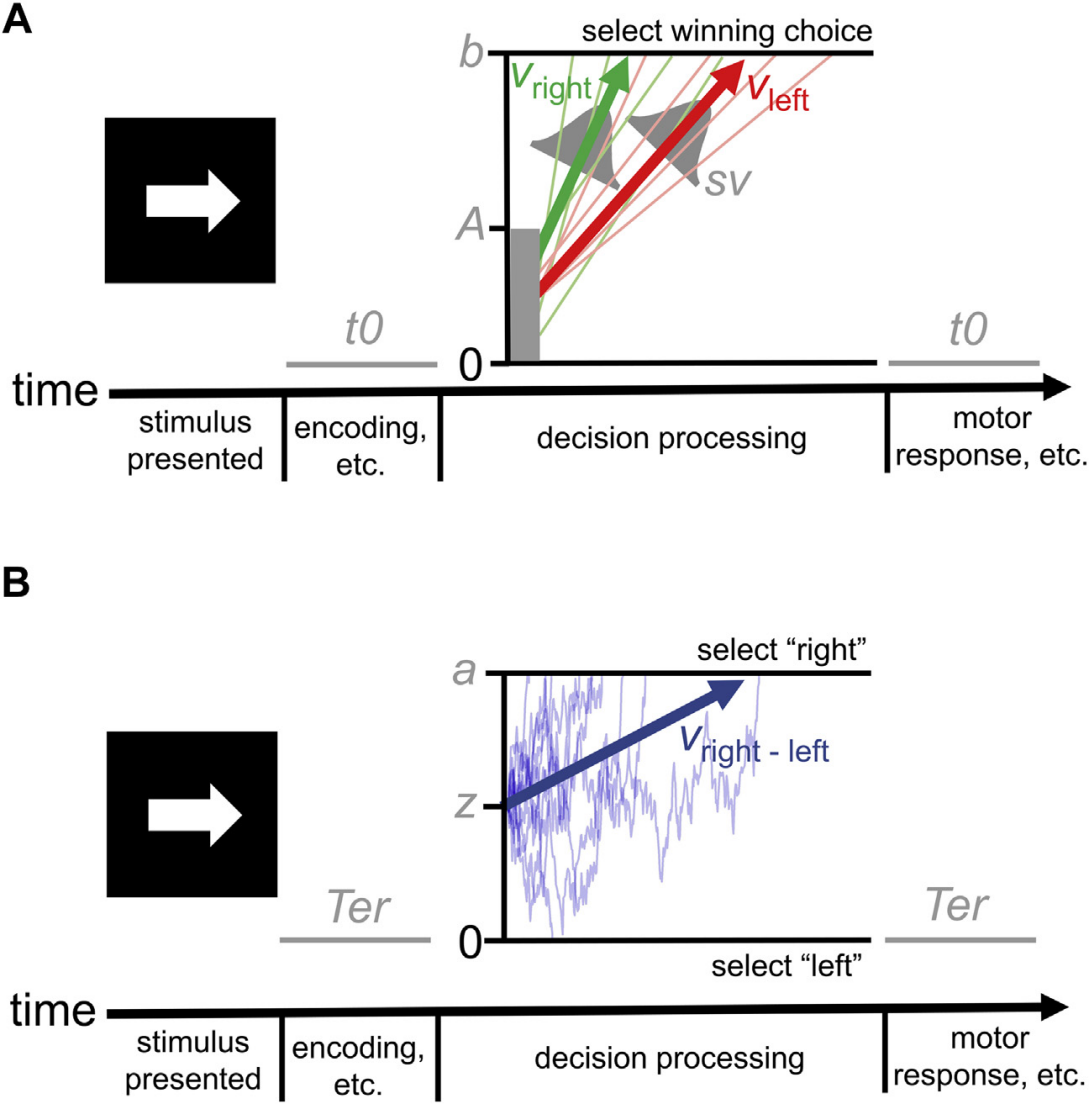
Schematics of the **(A)** linear ballistic accumulator (LBA) and **(B)** diffusion decision model (DDM), which are commonly applied sequential sampling models in the accumulator-type class and random-walk class, respectively. In both illustrations, the models describe a task in which an individual must decide whether a presented arrow is pointing to the left or the right. The LBA assumes that accumulators for the correct choice (right, in green) and incorrect choice (left, in red) start at a level drawn from a uniform distribution between 0 and parameter *A* and proceed to gather evidence at linear and deterministic rates over time as they race toward an upper response threshold, set at parameter *b*. The rates of evidence accumulation on individual trials, represented by the light green and light red traces, are drawn from normal distributions with a mean of *v* (represented by the green, *v*_*right*_, and red, *v*_*left*_, arrows) and a standard deviation of *sv*. The DDM instead assumes a single decision variable that represents the relative amount of evidence for each of the two possible choices (e.g., evidence for right vs. left; these models are typically applied to two-choice decisions). This variable begins at parameter *z* and drifts over time between boundaries for each possible response, set at 0 (for left) and parameter *a* (for right). The drift process on individual trials, represented by the light blue traces, is stochastic and moves toward the boundary for the correct choice at an average rate of *v* (represented by the blue arrow, *v*_*right–left*_). Efficiency of evidence accumulation, defined as the rate at which an individual is able to gather relevant evidence from the environment to make accurate choices, can be measured in the LBA by subtracting the average accumulation rate for the incorrect choice (*v*_*left*_) from that of the correct choice (*v*_*right*_). Efficiency of evidence accumulation is also measured by the DDM’s single average drift rate parameter (*v*_*right–left*_). Individuals’ level of response caution (i.e., speed/accuracy trade-off) can be indexed by parameters that represent the distance evidence accumulators must travel to trigger a response in both the LBA (parameter *b*) and DDM (parameter *a*). Both models also include parameters for time taken up by perceptual and motor processes peripheral to the decision, *t0* and *Ter*, respectively.

SSMs come in two general variants (62) (Figure 1). In accumulator-type models, such as the linear ballistic accumulator (LBA) (73), evidence is gathered by separate accumulators for each response option that race toward an upper threshold. In random-walk models, such as the diffusion decision model (DDM) (63,74), relative evidence for each choice is represented as a single total that drifts between boundaries representing each option. The DDM and LBA, which are the most widely used models in each class, differ on several major assumptions. Most prominently, the DDM assumes that the rate of evidence accumulation varies stochastically over time within a trial. Conversely, the LBA assumes that the evidence accumulates in a linear and deterministic manner and that any variability in accumulation rate occurs between trials. Although the within-trial variability of the DDM may be more biologically plausible, the LBA’s simplified assumptions do not appear to limit its descriptive power and make it easier to apply (73).

Despite these differences, parameters from both models can be used to measure three key latent processes: 1) the drift rate, or EEA; 2) the threshold or boundary separation, which reflects an individual’s level of caution (i.e., speed/accuracy trade-off); and 3) nondecision time, which accounts for the time spent on peripheral (e.g., motor) operations. Applications of the DDM and LBA to the same empirical data generally suggest similar conclusions about these three key processes (75,76), although process parameterization differs slightly between the models (Figure 1), and they sometimes offer divergent accounts of other constructs [e.g., variability in memory evidence (77)].

Several considerations are relevant when using these models. First, researchers should seek to ensure that the behavioral tasks analyzed respect SSM assumptions [e.g., number of processing stages, parameter invariance across time, and others detailed in (65)]. That said, recent work on complex paradigms has suggested that inferences from SSMs often remain robust despite violations of certain assumptions (78,79). Second, parameters that measure processes of interest must be able to be accurately estimated from empirical data (80). Small numbers of trials and greater model complexity (i.e., more parameters) impede parameter estimation, which may force investigators to select more parsimonious models. For example, several specialized SSMs have been proposed to explain processing on inhibition (e.g., Stroop) tasks (70,72), but parameters for these complex models are difficult to estimate at trial numbers common in empirical studies (81). Therefore, an alternative approach [e.g., (25,30)] is to fit a standard DDM to these tasks under the assumption that measurement of the main processes of interest will be robust despite some misspecification in the simpler model.

The use of SSMs to describe and differentiate cognitive mechanisms has several benefits. First, SSMs posit detailed mechanistic accounts that explain how underlying cognitive operations produce observed patterns of behavior. Thus, they make specific, quantitative predictions about behavioral data (e.g., skew of response time distributions, slow vs. fast errors) that are generally well supported in a substantial literature (73,74,82). Second, mechanisms posited in these models have clear links to neurophysiological processes. Neural firing patterns recorded in nonhuman primates across multiple brain regions during decision making display properties consistent with evidence accumulation (62,83, 84, 85), and these patterns have recently been quantitatively linked to SSM parameters in joint neural and behavioral models (86,87). Hence, SSMs display clear evidence of biological plausibility, providing an important bridge between neurophysiology and human behavioral research. Third, SSMs allow selective measurement of latent cognitive mechanisms. Standard metrics derived from laboratory tasks, such as response time (RT) and accuracy, are influenced by confounding factors such as subjects’ preferences to prioritize speed versus accuracy. However, SSMs can recover precise estimates of critical parameters irrespective of subjects’ strategies (65). A recent simulation study suggests that SSMs’ ability to measure latent processes selectively (e.g., indexing cognitive efficiency independent of speed/accuracy preferences) boosts statistical power (88). Finally, as detailed below, SSMs are beginning to provide novel insights into the structure of individual differences in cognition across the spectrum of health and psychopathology.

## EEA as a Foundational Individual Difference Dimension

**Figure 2:**
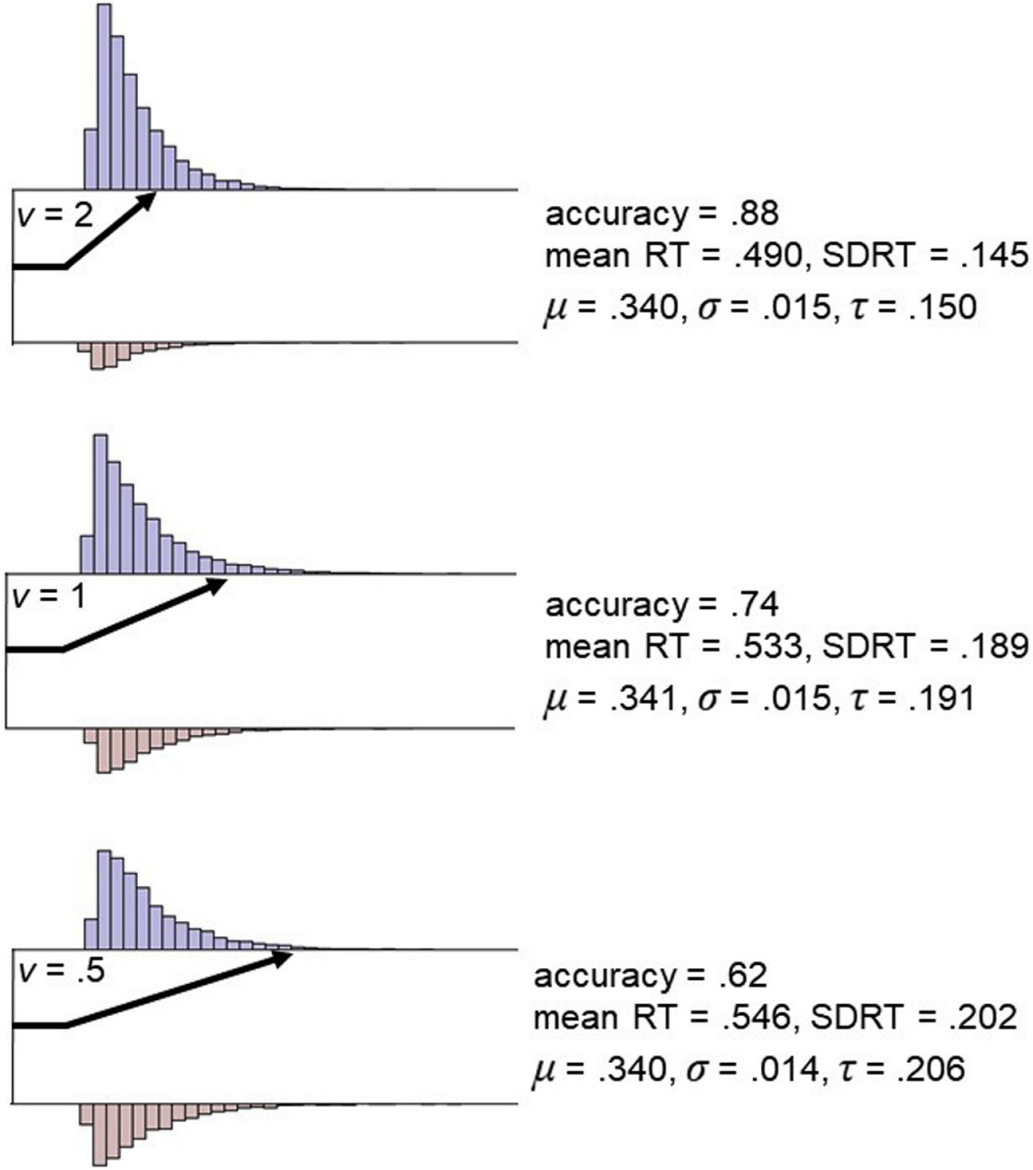
Simulated data that illustrate the behavioral manifestations of differences in efficiency of evidence accumulation (EEA). Response time (RT) data from 10,000 trials were simulated with the diffusion decision model implemented in the R package rtdists (149) while varying drift rate (*v* = 2, 1, 0.5) and holding other diffusion decision model parameters constant (*a* = 1, *z* = 0.5, *Ter* = 0.300). Blue histograms represent simulated correct RTs, while red histograms represent simulated error RTs. As EEA (*v*) decreases, accuracy rates are reduced and both the mean and standard deviation of RT increase. However, analysis of RTs with the ex-Gaussian distribution, a statistical model that allows Gaussian and exponential components of RT distributions to be indexed separately, reveals that the mean (*μ*) and Gaussian variability (*σ*) stay relatively constant, while exponential RT variability (*τ*; positive skew) substantially increases at lower levels of EEA. Therefore, as demonstrated in previous large-scale simulation studies (91), EEA primarily affects RT distributions’ level of exponential RT variability, with larger *τ* estimates (i.e., greater levels of positive skew) providing a behavioral hallmark for reduced EEA.

A burgeoning individual differences literature [reviewed in detail in (89,90)] has begun to demonstrate SSMs’ utility for characterizing fundamental mechanisms of cognition. This work has primarily focused on the DDM’s drift rate parameter, which indexes EEA, or the rate at which an individual gathers relevant evidence from the environment to make accurate choices in the context of background noise. Simulated DDM data in Figure 2 illustrate the behavioral consequences of variation in EEA; lower drift rates lead to lower accuracy and greater RT variability, primarily by increasing the positive skew of RT distributions (91).

**Figure 3:**
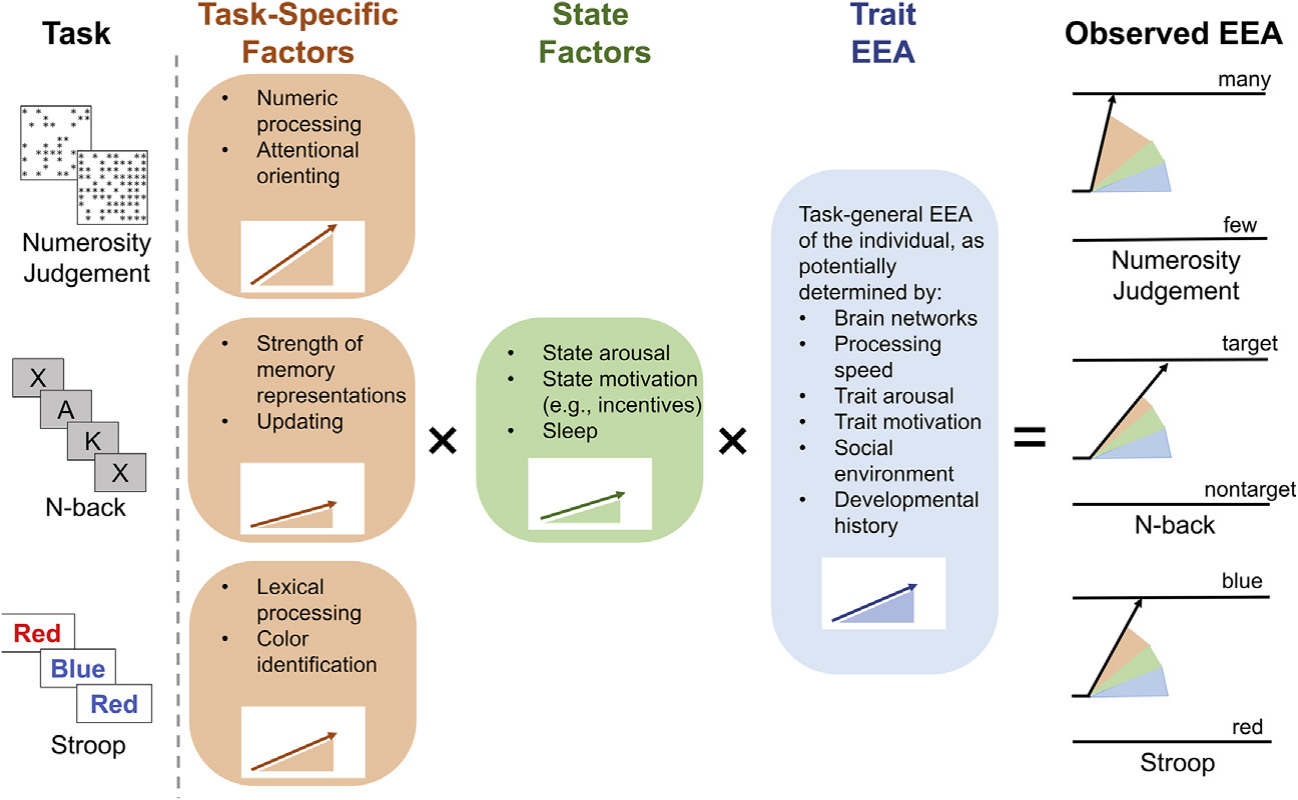
Hypothesized determinants of efficiency of evidence accumulation (EEA) manifested on specific cognitive tasks for a given individual.

Observed EEA for an individual on a given cognitive task is likely the product of multiple processes (Figure 3). Although task-specific mechanisms (e.g., individuals’ color identification ability on the Stroop) and state factors [e.g., motivation (92, 93, 94, 95)] may play key roles, a growing body of findings suggests that a large portion of the variance in EEA is explained by a domain-general, trait-like factor. EEA estimates from choice tasks across different cognitive domains show strong correlations with one another, allowing the formation of a domain-general latent variable (96, 97, 98, 99, 100, 101), and recent work demonstrates that this general factor remains present even after explicitly accounting for domain-specific variance in EEA (78). EEA estimates are test-retest reliable under ideal measurement conditions [e.g., 200–400 trials (102)], and work using latent state-trait modeling across an 8-month interval found that state-related variance in EEA measures was statistically indistinguishable from zero, while trait-related variance was close to that found for intelligence tests (44% on average) (96). As EEA measured via relatively simple choice tasks correlates strongly with EEA on more complex paradigms and predicts better working memory ability and intelligence (78,97, 98, 99, 100, 101,103, 104, 105), trait EEA may be a critical determinant of individual differences in higher-order cognitive abilities. Taken together, this body of work indicates that EEA is a psychometrically robust cognitive individual difference dimension that appears to be foundational to the performance of a wide variety of tasks. Importantly, the fact that trait EEA is derived from a formal, mechanistic theory of the data-generating process across cognitive measures contrasts with constructs in the fractionation paradigm, which are not linked to a generally applicable mechanistic theory.

## Reduced EEA as a Transdiagnostic Neurocognitive Risk Factor for Psychopathology

The behavioral signatures of reduced EEA—variable RTs and less accurate responding—have long been documented in the task performance of individuals with diverse psychiatric diagnoses (95,106, 107, 108, 109, 110). Yet, SSMs have only recently been applied in the context of clinical research. Because RT variability has been of longstanding interest in ADHD (95), SSMs have been most extensively used to study this disorder. As reviewed by others (95,111,112) and supported by subsequent work (104,113, 114, 115, 116, 117), individuals with ADHD consistently display reduced EEA in SSM analyses, and meta-analytic effect size estimates for comparisons with healthy participants are in the moderate to large range: *d* = 0 .75 (112) and *g* = 0.63 (95). What is arguably most striking about these effects is the breadth of domains in which EEA reductions are observed, including simple perceptual decision making (105,114,118), sustained attention (112,117,119), inhibition (120, 121, 122, 123), pattern learning (116,124), and interval timing (115). Furthermore, stimulant medication treatments for ADHD have been found to improve EEA in both children with the disorder (92) and healthy adults (125), suggesting that EEA could help mediate treatment effects. In the latter study (125), stimulants enhanced EEA similarly in an incongruent task condition (thought to engage executive control) and a congruent task condition (where control is thought to be unengaged). Taken together with the pattern of cross-task effects observed in ADHD, this finding suggests that both ADHD-related deficits and treatment-related improvements in EEA are domain general, spanning diverse tasks and conditions with varying levels of complexity and executive demands.

Beyond ADHD, reduced EEA has been documented in schizophrenia (126,127), depression (128), and individuals at risk for frequent substance use (129). Extending these findings, our recent work has provided evidence that EEA is a transdiagnostic risk factor for psychopathology (130). In a large sample drawn from the UCLA Consortium for Neuropsychiatric Phenomics (131) we found that a latent EEA factor derived from multiple tasks was substantially reduced in patients with ADHD, schizophrenia, and bipolar disorder relative to healthy participants (*d* = 0.51, 1.12, and 0.40, respectively) and displayed a negative correlation with the overall severity of individuals’ cross-disorder psychopathology symptoms (*r* = −.20). As this study made the simplifying assumption, discussed above—that the standard DDM can provide adequate measures of EEA on inhibition tasks—replication of these results using more complex modeling procedures is warranted.

We now present a hypothesis that seeks to build on this growing array of observations. We posit that lower trait EEA conveys broad risk for psychopathology and that EEA can therefore account for a substantial proportion of performance decrements on tests of neurocognitive abilities that are observed across psychiatric disorders. Moreover, we propose that reductions in EEA similarly impair performance across tasks of varying levels of complexity, rather than selectively impacting executive tasks. These claims are rendered plausible by the research reviewed above documenting that 1) trait EEA displays clear validity as a task-general cognitive individual difference dimension; 2) in healthy individuals, EEA explains a large portion of the variance in higher-order cognitive functioning; 3) EEA is impaired across multiple psychopathologies; 4) EEA impairments are present across a wide range of cognitive paradigms; and 5) individuals with psychiatric diagnoses linked to neurocognitive decrements, such as ADHD and schizophrenia, have been found to display such decrements across both complex executive tasks and simple choice RT paradigms.

Although we view this evidence as compelling, we note that direct tests of our hypothesis, which have yet to be completed, would require several features. First, these tests would require that large and demographically diverse samples of individuals with and without psychiatric diagnoses complete batteries of tasks that can be used to accurately estimate SSM parameters. Second, because precise measurement of trait EEA requires latent variables informed by performance in multiple domains (78,90), tasks would need to span cognitive processing modalities (e.g., verbal, numeric) and the executive/nonexecutive continuum. Such data would allow the derivation of latent trait EEA metrics and assessments of EEA’s relations with an array of disorders and psychopathology symptoms.

We also note three important qualifications to our claims. First, the task generality of trait EEA does not imply that the computational processes involved in the execution of tasks from diverse cognitive domains are identical. Rather, the psychometric work reviewed above indicates that trait EEA is a primary factor driving individual differences (and therefore, we suspect, clinical differences) in task performance. Although different tasks require cognitive operations involving distinct types of evidence (Figure 3), the fact that SSMs provide a highly generalizable account of processing across tasks suggests that task-general mechanisms involved in accumulation of multiple types of evidence could plausibly drive individual differences in EEA. Indeed, estimates of task-specific variance in EEA from state-trait models are strikingly low (≤17%) (96). Second, we do not claim that trait EEA is itself determined by a single underlying process. As we outline below, current evidence suggests that EEA is likely influenced by an array of biological and contextual factors. EEA may thus serve as a “watershed node” (132) in a complex matrix of causation. Watersheds are shaped by multitudinous converging water channels, but once formed, they are subsequently relatively unitary drivers of downstream effects. Similarly, we propose that EEA has multifactorial determinants but serves as a relatively unitary driver of cognitive deficits and clinical symptoms. Third, although we posit that EEA is a prominent contributor to psychopathology-related deficits on tests of cognitive abilities, it is almost certainly the case that a much broader array of factors, beyond EEA and beyond other influences on cognitive test performance, contribute to individuals’ psychopathology symptoms. Unlike EEA, other contributors to psychopathology may be difficult to capture on laboratory cognitive tasks and may therefore be better measured with alternative methods (e.g., questionnaires, biomarkers).

**Figure 4:**
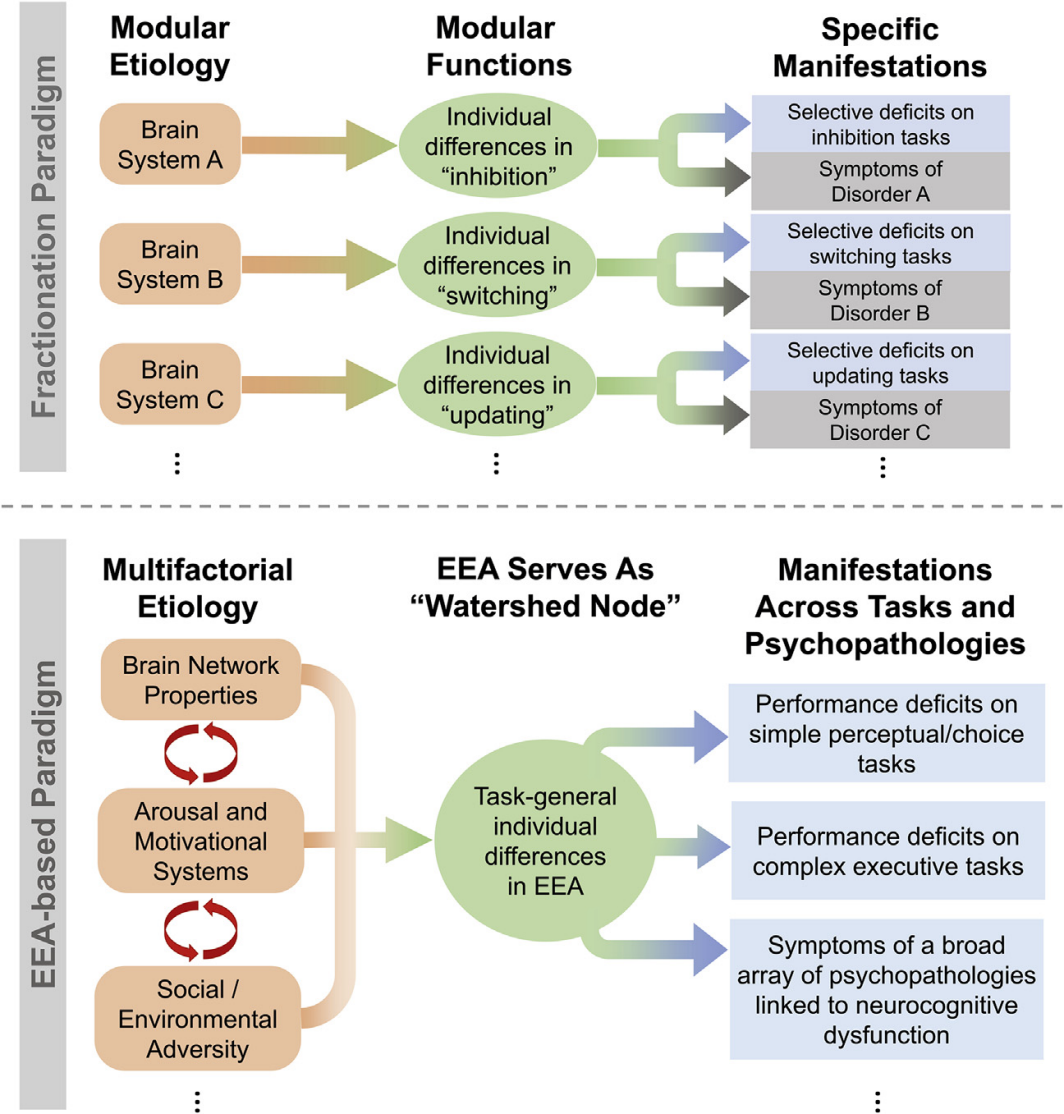
Diagrams contrasting the general assumptions of two different approaches to studying neurocognitive contributions to psychopathology: the dominant fractionation paradigm (top) and the alternative efficiency of evidence accumulation (EEA)–based paradigm we highlight in this review (bottom).

The overall framework we propose is outlined and contrasted with the conventional fractionation framework in Figure 4. We now examine its broader implications.

## Implications of an EEA-Based Computational Framework for Clinical Neuroscience

### A Focus on Complex Executive Tasks May Be Misplaced

The preoccupation of psychiatric cognitive neuroscience with response inhibition and other executive constructs is understandable. There are clearly clinically important individual differences in the ability to resist cravings for an addictive substance or to regulate tendencies to mind wander during a boring lecture. Because tasks such as the Stroop were designed to selectively isolate top-down control, it makes sense that these tasks are seen as key elements of research into regulatory problems in psychopathology. However, the evidence reviewed above suggests that these tasks are not, in fact, selectively isolating executive processes.

The alternative possibility we put forward is that aberrant performance on complex executive tasks in psychopathology largely reflects task-general reductions in EEA. If this view is correct, it follows that the field’s focus on executive functions, and the experimental paradigms thought to measure them, is overly narrow. To better understand the ability to attend to a lecture or resist cravings, it may be more fruitful to investigate the clinical correlates and neural basis of task-general impairments in EEA. At the level of study design, cross-domain batteries of relatively simple cognitive tasks that are optimized for computational modeling (e.g., perceptual choice) may be as good as, or preferable to, complex tasks that attempt to experimentally isolate executive processes.

### Subtraction in Cognitive and Neuroimaging Measures Is Counterproductive

EEA’s potential role in task-general deficits similarly calls into doubt the use of subtraction methods that attempt to isolate individual differences in specific neurocognitive processes (e.g., contrasting behavior or neural activation in conditions that do and do not require inhibition). If EEA is the primary driver of individual differences in performance across task conditions, subtraction likely obscures, rather than enhances, measurement of the clinically relevant process.

Recent findings support this notion. In a large nonclinical sample (30), we found that subtraction-based metrics show negligible relations across tasks and do not predict self-report indices of self-control (133). Nonetheless, EEA estimates across these same tasks, and across executive/nonexecutive conditions (again obtained under the simplifying assumption that the standard DDM can adequately index EEA from inhibition paradigms), formed a coherent latent factor that was related to self-regulation (*r* = .18) (133). Similarly, in a neuroimaging study of the go/no-go task (134), EEA estimated from trials that require inhibition (no-go) was strongly correlated (*r* = .73) with EEA on trials that do not (go), suggesting that performance across conditions was largely determined by a single dimension (134). At the neural level, activation from the commonly used neuroimaging contrast that subtracts activity during go trials from activity during correct no-go trials displayed little evidence of relationships with performance metrics (including EEA), questioning the utility of subtraction for neural measures (134). Hence, clinical neuroscientists may be better off focusing on commonalties across cognitive task conditions than on differences between them.

### Findings of Disorder-Specific Deficits in Neurocognitive Test Data May Be Elusive

A related implication is that efforts to use neurocognitive test data to identify deficits in specific cognitive functions that differentiate disorders (e.g., inhibition in ADHD) may face significant challenges. Indeed, our hypothesis that EEA is a primary driver of individual differences in performance across commonly used tasks provides an explanation for the already well-acknowledged failure of such tasks to characterize selective deficits for many disorders (3,15,43, 44, 45, 46, 47, 48, 49). Although some may view this conclusion as discouraging, we believe that it fits with emerging views of psychopathology that emphasize transdiagnostic individual difference dimensions (e.g., Research Domain Criteria and Hierarchical Taxonomy of Psychopathology) (17,135), in which positions on multiple such dimensions characterize disorders. Specifically, it is likely that EEA, as measured on neurocognitive tasks, is one of many relevant transdiagnostic dimensions and must be combined with indices of constructs derived from other measurement domains (e.g., socioemotional, biological) to better characterize variation in, and the multifactorial causes of, psychopathology.

### EEA Can Provide a Window Into the Basis of Neurocognitive Deficits in Psychopathology

A shift in psychiatric cognitive neuroscience research focus toward EEA is likely to produce novel mechanistic insights and facilitate translation across behavioral, systems, and neurophysiological levels of analysis. As outlined above, a major advantage of using SSMs to guide research is that the evidence accumulation processes they posit are not only biologically plausible but well supported by extant neurophysiological research in nonhuman primates (62,83, 84, 85, 86,136). Corresponding neural signatures of these processes in humans have also been well characterized with electroencephalography (137, 138, 139) and functional magnetic resonance imaging (140, 141, 142, 143). Although these signatures are distributed throughout multiple cortical areas, there is converging evidence that the frontoparietal network and anterior insula play especially important roles (144).

Research on the neural basis of trait EEA is sparser. A small number of studies using disparate methodologies have linked between-individual differences in EEA to parietal activation during decision making (145), salience network responses to errors (134), and greater structural and functional connectivity in the frontoparietal network (146). However, these studies are limited by their measurement of EEA with individual tasks, rather than with the recommended cross-domain latent factors (90). Findings that EEA is enhanced by catecholamine agonists (92,125,147) indicate that EEA may be related to the integrity of dopamine or norepinephrine systems. Incentives also alter EEA (92, 93, 94), suggesting that stable traits related to motivational processes [e.g., cognitive effort discounting (148)] could affect how individuals react to these state-related factors during cognitive performance. We do not offer a comprehensive hypothesis about the etiology of individual and clinical differences in EEA because we believe doing so would be premature. However, strong evidence for the existence of a task-general trait EEA factor suggests that broad neurobiological and/or contextual (e.g., poverty) influences could affect cognitive performance though EEA.

The study of individual differences in EEA could usher in a new paradigm for understanding cognitive abnormalities in psychopathology. Rather than attempting to fractionate putative disorder-specific deficits, this paradigm would instead focus on how EEA is determined by neurophysiological processes, neurotransmitter systems, brain networks, and contextual factors such as motivation, stress, and social adversity. Doing so would move the study of these influences on disordered cognition into a more mechanistic computational framework.

## Conclusions

This review assessed the emerging literature on the application of mathematical process models to the study of individual and clinical differences in neurocognition. We argue that this literature presents a compelling case that trait EEA, a foundational individual difference dimension formally defined in computational models, is likely a primary driver of observed deficits on tests of neurocognitive abilities across clinical disorders. Adopting an EEA-focused research approach has the potential to transition clinical neuroscience away from measures that have poor psychometric properties and constructs that are biologically amorphous. In contrast, EEA is a precisely defined construct that has strong psychometric properties, displays clear links to psychopathology, and is well positioned to yield richer connections with neurobiology.
